# Extensive *In Silico* Analysis of *ATL1* Gene : Discovered Five Mutations That May Cause Hereditary Spastic Paraplegia Type 3A

**DOI:** 10.1155/2020/8329286

**Published:** 2020-04-19

**Authors:** Mujahed I. Mustafa, Naseem S. Murshed, Abdelrahman H. Abdelmoneim, Miyssa I. Abdelmageed, Nafisa M. Elfadol, Abdelrafie M. Makhawi

**Affiliations:** ^1^Department of Biotechnology, University of Bahri, Khartoum, Sudan; ^2^Department of Microbiology, International University of Africa, Khartoum, Sudan; ^3^Faculty of Medicine, Alneelain University, Khartoum, Sudan; ^4^Department of Pharmacy, University of Khartoum, Khartoum, Sudan; ^5^Department of Microbiology, National Ribat University, Khartoum, Sudan

## Abstract

**Background:**

Hereditary spastic paraplegia type 3A (SPG3A) is a neurodegenerative disease inherited type of Hereditary spastic paraplegia (HSP). It is the second most frequent type of HSP which is characterized by progressive bilateral and mostly symmetric spasticity and weakness of the legs. SPG3A gene mutations and the phenotype-genotype correlations have not yet been recognized. The aim of this work was to categorize the most damaging SNPs in *ATL1* gene and to predict their impact on the functional and structural levels by several computational analysis tools.

**Methods:**

The raw data of *ATL1* gene were retrieved from dbSNP database and then run into numerous computational analysis tools. Additionally; we submitted the common six deleterious outcomes from the previous functional analysis tools to I-mutant 3.0 and MUPro, respectively, to investigate their effect on the structural level. The 3D structure of *ATL1* was predicted by RaptorX and modeled using UCSF Chimera to compare the differences between the native and the mutant amino acids.

**Results:**

Five nsSNPs out of 249 were classified as the most deleterious (rs746927118, rs979765709, rs119476049, rs864622269, and rs1242753115).

**Conclusions:**

In this study, the impact of nsSNPs in the ATL1 gene was investigated by various *in silico* tools that revealed five nsSNPs (V67F, T120I, R217Q, R495W, and G504E) are deleterious SNPs, which have a functional impact on ATL1 protein and, therefore, can be used as genomic biomarkers specifically before 4 years of age; also, it may play a key role in pharmacogenomics by evaluating drug response for this disabling disease.

## 1. Introduction

Hereditary spastic paraplegia type 3A (SPG3A) is a neurodegenerative disease inherited type of Hereditary spastic paraplegia (HSP) [1]. SPG3A is the second most frequent type of HSP [2–4]. SPG3A is characterized by muscle stiffness with paraplegia and early onset of symptoms [5, 6]; however, in rare cases, extremely severe characteristic with neonatal onset has been reported [6]. SPG3A had been recognized in different populations [7–10].

Insight into the molecular basis of HSP is increasing rapidly; different genes cause clinically indistinguishable HSP types [11–13]. SPG3A is triggered by heterozygous mutations in *ATL1* gene; *ATL1* is a protein coding gene which is located in chromosome 14 at position 22 in the forward strand (14q22.1). It provides instruction for production of atlastin-1 protein, which is a member of guanosine triphosphatases (GTPases) family. It is primarily produced in the brain and the spinal cord in the central nervous system. In neurons, this protein is located mainly in the endoplasmic reticulum and it is responsible for endoplasmic reticulum tubular network biogenesis; also, it is located at the tip of neurons in the axonal growth cone, it directs the growth and development of the axons which transmit nerve impulses [14–18]. Mutations in the *ATL1* gene are more common with early-onset HSP patients. [19] These mutations include numerous missense mutations, particularly at the guanosine-binding domain [7]. Small insertion and deletion as well as whole exon deletion, but until now, the correlation of genotype phenotype is not clear [20]. *ATL1* gene mutations likely disrupt neuron function, and it leads to impairment of the distribution of materials within these cells and restriction in the growth of axons of neurons. These problems can lead to abnormal functioning or death of the neurons in the corticospinal tracts. As a result, they will become unable to transmit nerve impulses, particularly to other neurons and muscles in the lower extremities. This impaired function leads to the signs and symptoms of spastic paraplegia type 3A.

The population databases (http://exac.broadinstitute.org/) claim a rare benign SNPs also found in *ATL1* in healthy persons. Besides, a rare mutation in the *ATL1* has been reported in axonal motor neuropathy [17, 21] and hereditary sensory neuropathy type I patients [22]. Most importantly, given the absence of cure, it is vital for appropriate genetic counseling for the manifestation of any variants related to SPG3A because the treatment is symptomatic; muscle stiffness can be treated with oral baclofen or tizanidine; physical therapy should be combined to improve the life quality of the patient [1].

The problematic issue stems from the point that the effects of genetic differences on macromolecules function vary extensively making it difficult to decode genotype-phenotype correlation; some studies suggest that linkage analysis is a beneficial approach to assist in understanding this correlation. [23].

This is the first translational bioinformatics analysis in the coding region of *ATL1* gene which aims to categorize nsSNPs that could be used as genomic biomarkers specifically before 4 years of age; also, it may play a key role in pharmacogenomics by evaluating drug response for this disabling disease [24–29].

## 2. Methods

### 2.1. Data Mining

The data of the *ATL1* gene were retrieved from dbSNP database, (http://www.ncbi.nlm.nih.gov/snp/), while the protein reference sequence was obtained from UniProt database (https://www.uniprot.org/).

### 2.2. Functional Analysis

#### 2.2.1. SIFT

SIFT was used to observe the effects of each amino acid substitution on protein function. SIFT predicts damaging SNPs on the basis of the degree of conserved amino acid residues in aligned sequences to the closely related sequences, gathered through PSI-BLAST. [30].

#### 2.2.2. PolyPhen-2

PolyPhen-2 stands for polymorphism phenotyping version 2. We used PolyPhen-2 to study potential effects of each amino acid substitution on structural and functional properties of the protein by considering physical and comparative approaches. The input data needs accession number and position of mutations and native and altered amino acids [31].

#### 2.2.3. PROVEAN

It predicts whether an amino acid substitution has an effect on the biological function of a protein grounded on the alignment-based score. If the PROVEAN score is ≤−2.5, the protein variant is predicted to have a “deleterious” effect, while if the PROVEAN score is >−2.5, the variant is predicted to have a “neutral” effect [32].

#### 2.2.4. SNAP2

It is a trained functional analysis web-based tool that differentiates between effect and neutral SNPs by taking a variety of features into account. SNAP2 has an accuracy (effect/neutral) of 83%. It is considered an important and substantial enhancement over other methods [33].

#### 2.2.5. SNPs&GO

It is a support vector machine (SVM) based on the method to accurately predict the disease-related mutations from protein sequence. The probability score higher than 0.5 reveals the disease-related effect of mutation on the parent protein function. [34].

#### 2.2.6. PHD-SNP

It is an online Support Vector Machine- (SVM-) based classifier, which is optimized to predict if a given single-point protein mutation can be classified as disease-related or as a neutral polymorphism. [35].

### 2.3. Stability Analysis

#### 2.3.1. I-Mutant 3.0

Change in protein stability disturbs both protein structure and protein function. I-Mutant is a suite of support vector machine. It offers the opportunity to predict the protein stability changes upon single-site mutations. The FASTA format sequence of ATL1 protein was retrieved from UniProt that is used as an input to predict the mutational effect on protein and stability RI value (reliability index) computed. [36].

#### 2.3.2. MUPro

It is a support vector machine-based tool for the prediction of protein stability changes upon nonsynonymous SNPs. The value of the energy change is predicted, and a confidence score between -1 and 1 for measuring the confidence of the prediction is calculated. A score <0 means the variant decreases the protein stability; conversely, a score >0 means the variant increases the protein stability. [37].

### 2.4. Biophysical and Visualization Analysis

#### 2.4.1. Project HOPE

It is a server to search structural data from several databases such as UniProt. The FASTA format sequence of ATL1 protein was retrieved from UniProt that is used as an input to predict the biophysical validation for our SNPs of interest. The main aims for the submissions in Project HOPE are to analyse and confirm the results that we had obtained earlier [38].

#### 2.4.2. RaptorX

The full 3D structure of human ATL1 protein is not available in the Protein Data Bank. Hence, we used RaptorX to generate a 3D structural model for wild-type ATL1. The FASTA format sequence of ATL1 protein was retrieved from UniProt that is used as an input to predict the 3D structure of human ATL1 protein. [39].

#### 2.4.3. UCSF Chimera

UCSF Chimera is a highly extensible program for interactive visualization and analysis of molecular structures and related data, including density maps, supramolecular assemblies, sequence alignments, docking results, and conformational analysis Chimera (version 1.8) [40].

#### 2.4.4. Ramachandran Plot Analysis

Geometrical validation through the Ramachandran plot provides a single measure encapsulating the major structure-validation information contained in bond angle distortions. We used the BDP model for ALT1 protein we acquired from RaptorX and submitted it to this software, and then we downloaded the resulted predictions [41]. We also used the model plane command from Chimera software to visualize the Ramachandran plot.

### 2.5. ConSurf Server

It is a web server which suggests evolutionary conservation reviews for proteins of known structure in the PDB. ConSurf detects the similar amino acid sequences and runs multialignment approaches. The conserved amino acid across species detects its position using specific algorithms. [42].

### 2.6. GeneMANIA

It is a web server creating proposition about gene function, investigating genelists and prioritizing genes for functional assays. The high accuracy of the GeneMANIA prediction algorithm and large database make the GeneMANIA a useful tool for any biologist. [43].

### 2.7. ClinVar

It is a public archive of reported studies of the relationships among human variations and phenotypes, with supporting evidence. We used it to compare our predicted approach with the clinical one [44].

### 2.8. Variant Effect Predictor (VEP)

The Ensembl Variant Effect Predictor software provides toolsets for an organized approach to annotate and assist for prioritization of mutations. The input data format was a list of variant identifiers, while the output was filtered by choosing 1000 genome combined population to expend the population coverage [45].

## 3. Results

**Figure 1:**
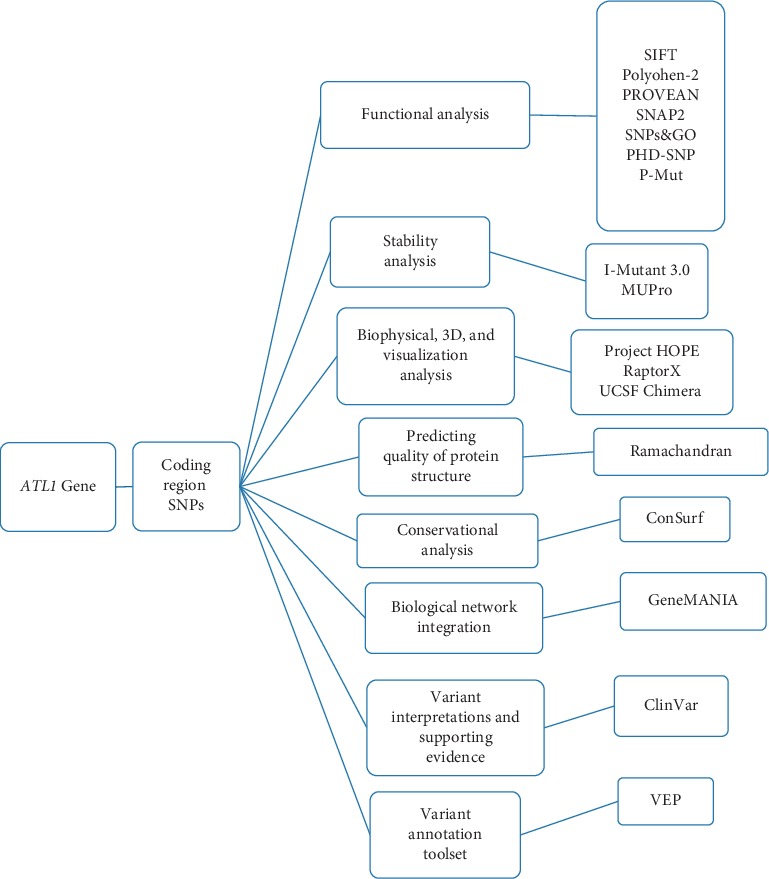
Graphic demonstration of *ATL1* gene work flow.

The total number of SNPs in the coding region that were recovered from NCBI was 249 nsSNPs, and these SNPs were submitted into different functional analysis online tools, (Figure 1) Ninety-eight out of 249 nsSNPs were found to be affected by SIFT, and 118 damaging SNPs (44 possibly damaging and 74 probably damaging) by Pholyphen-2 and 122 were found to be deleterious by PROVEAN; the triple-positive damaging SNPs were filtered from the earlier three online tools; out of 41 SNPs, there were 8 predicted damaging by SNAP2. (Table 1) After second filtration, the number of SNPs decreased to 8 and then were submitted into SNPs&GO and PhD-SNP and P-Mut to give more accurate results on their effect on the functional impact; the triple positive in the three tools was five SNPs (Table 2); on the other hand, the stability analysis on these five SNPs were tested by I-Mutant3.0 and MUPro, and the stability analysis revealed that all five SNPs decrease the protein stability, except for one SNP (T120I) that was predicted by I-Mutant3.0 to increase protein stability. (Table 3). We also analyzed the structural integrity of the predicted protein through the Ramachandran plot analysis, which shows 96.2 % of the residues to be in the favored region (Table 4).

## 4. Discussion

The in vitro approach is consuming a lot of time and fees which may or may not have a positive result from the study; on the other hand, the computational approach is entirely different; it saves time with low cost and gives rapid results to improve our understanding of how variants could interrupt the protein structure and function [46, 47].

Missense mutations are frequently found to arise at evolutionarily conserved regions. Those have a key role at structural and functional levels of the protein. [48–50] Therefore, our *in silico* analysis was devoted to the coding region of *ATL1* gene, which uncovered five disease-causing mutations that may cause SPG3A. The five deleterious SNPs come after extensive computational analysis, and seven online tools (Figure 1) were used to investigate the effect of each SNP on the functional impact; the reason why results are different in many times is because they run by different sequences and structure-based algorithms; (Tables 1 and 2) while two online tools (I-Mutant and MUPro) were used to investigate the effect of each SNP on the stability impact, the analysis revealed that all five SNPs decrease the protein stability, except for one SNP (T120I) that was predicted by I-Mutant3.0 to increase protein stability, (Table 3) thus proposing that these variants could destabilize the amino acid interactions causing functional deviations of protein to some point.

All these SNPs (V67F, T120I, and G504E) were recovered from the dbSNP as untested and all were found to be deleterious mutations; while these SNPs (R217Q and R495W) were recovered as pathogenic which agrees with our finding (Table 2).

**Figure 2:**
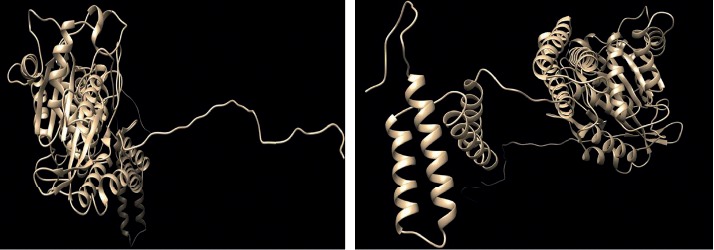
The 3D structure of the ATL1 protein model by two angles.

**Figure 3:**
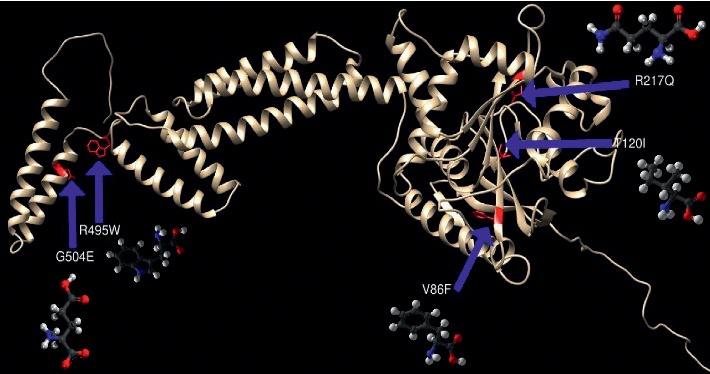
The 3D structure of the ATL1 protein model with the mutant amino acids.

**Figure 4:**
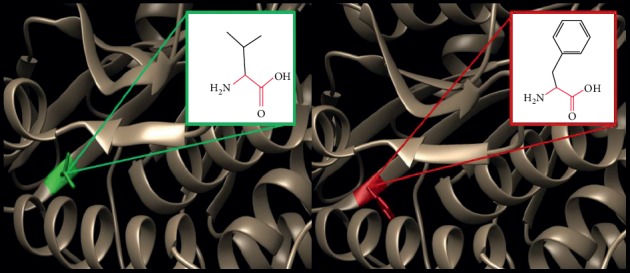
(V67F) : The amino acid Valine changes to Phenylalanine at position 67.

In order to investigate the biophysical properties of these variants, Project HOPE server was used to serve this purpose; RaptorX was used to create a 3D structure model for ATL1 protein, (Figure 2), while UCSF Chimera was used to visualize the amino acids change (Figure 3); in (Figure 4): (V67F) : ,the amino acid Valine changes to phenylalanine at position 67; the mutated residue is located in a domain that is important for binding of other molecules and in contact with residues in a domain that is important for the activity of the protein. The mutation might affect this interaction and thereby disturb signal transfer from the binding domain to the activity domain. The mutation introduces an amino acid with different properties, which can disturb this domain and abolish its function; the wild-type residue is very conserved, but the mutant residue is located near a highly conserved position.

**Figure 5:**
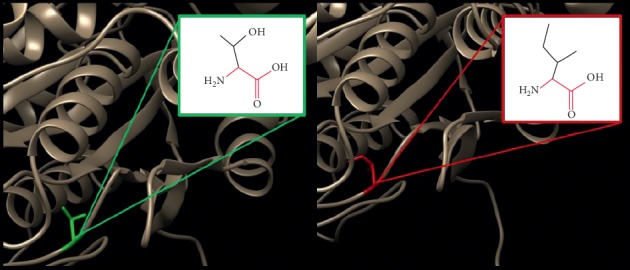
(T120I) : The amino acid Threonine changes to Isoleucine at position 120.

In (Figure 5): (T120I) : the amino acid threonine changes to isoleucine at position 120; the mutant residue is bigger than the wild-type residue, and these differences disturb the interaction with the metal-ion (MG); These differences in properties between wild-type and mutant residue can easily cause loss of interactions with the nucleotide (GTP), which can directly affect the function of the protein. The mutation is located within a domain, annotated in UniProt as (GB1/RHD3-type G). Only this residue type was found at this position. Mutation of a 100% conserved residue is usually damaging for the protein.

**Figure 6:**
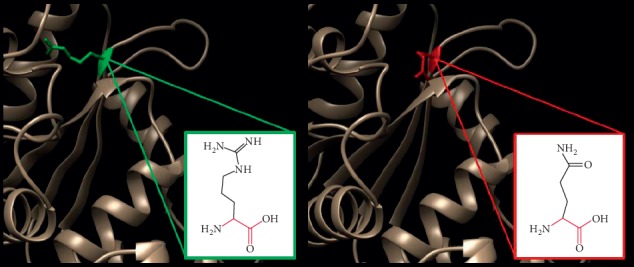
(R217Q) : The amino acid Arginine changes to Glutamine at position 217.

In (Figure 6): (R217Q) : the amino acid arginine changes to glutamine at position 217; in the 3D structure, the wild-type residue has interactions with a ligand annotated as GDP. The difference in properties between wild-type and mutation can easily cause loss of interactions with the ligand, because ligand binding is often important for the protein's function, and this function might be disturbed by this mutation. The wild-type residue charge was positive, while the mutant residue charge is neutral; the difference in charge will disturb the ionic interaction made by the original, wild-type residue. The mutated residue is in contact with residues in another domain. It is possible that the mutation disturbs these contacts and as such affect the protein function.

**Figure 7:**
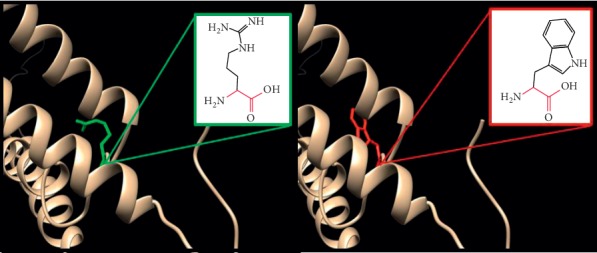
(R495W) : The amino acid Arginine changes to Tryptophan at position 495.

In (Figure 7): (R495W) : the amino acid arginine changes to tryptophan at position 495; the mutation is located within a stretch of residues annotated in UniProt as a special region, sufficient for membrane association. The differences in amino acid properties can disturb this region and disturb its function. The wild-type residue charge was positive, the mutant residue charge is neutral, and the loss of the charge can cause loss of interactions with other molecules or residues; the mutation introduces a more hydrophobic residue at this position. This can result in loss of hydrogen bonds and/or disturb correct folding.

**Figure 8:**
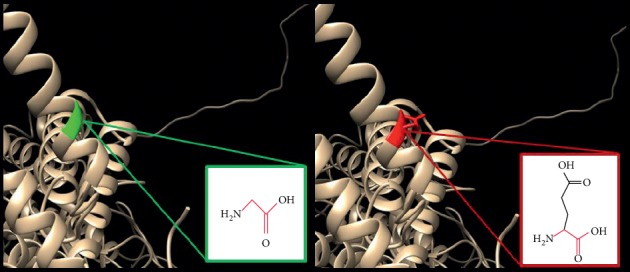
(G504E) : The amino acid Glycine changes to Glutamate at position 495.

In Figure 8, (G504E) : the amino acid *Glycine* changes to glutamate at position 495; the wild-type residue is a glycine, the most flexible of all residues. This flexibility might be necessary for the protein's function. Mutation of this glycine can abolish this function. The wild-type residue charge was neutral, the mutant residue charge is negative, and this can cause repulsion of ligands or other residues with the same charge. The torsion angles for this residue are unusual. Only glycine is flexible enough to make these torsion angles, and mutation into another residue will force the local backbone into an incorrect conformation and will disturb the local structure.

**Figure 9:**
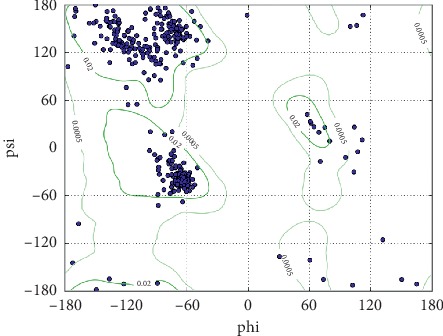
Ramachandran plot analysis of ATL1 protein showing most of the torsion angles located at the allowed region (the blue dots represent torsion angles; the green lines indicate the allowed region). (phi) *ϕ* and (psi) ψ are torsion angles. The torsion angle about the N—C bond is called *ϕ* and that about the C—C bond is ψ. This analysis is predicted by UCSF Chimera version 1.10.2.

The structure predicted through algorithm and software needs to be validated through other software like the Ramachandran plot analysis, which measures the accuracy of the structured data [51]. In our case 96.2 % of the residues are located in the favored region, which greatly validate our predicted structure (Table 4). Furthermore, Chimera software predictions showed most torsion angles to be located in the allowed regions which indicate that this is a good-quality protein structure (Figure 9).

**Figure 10:**
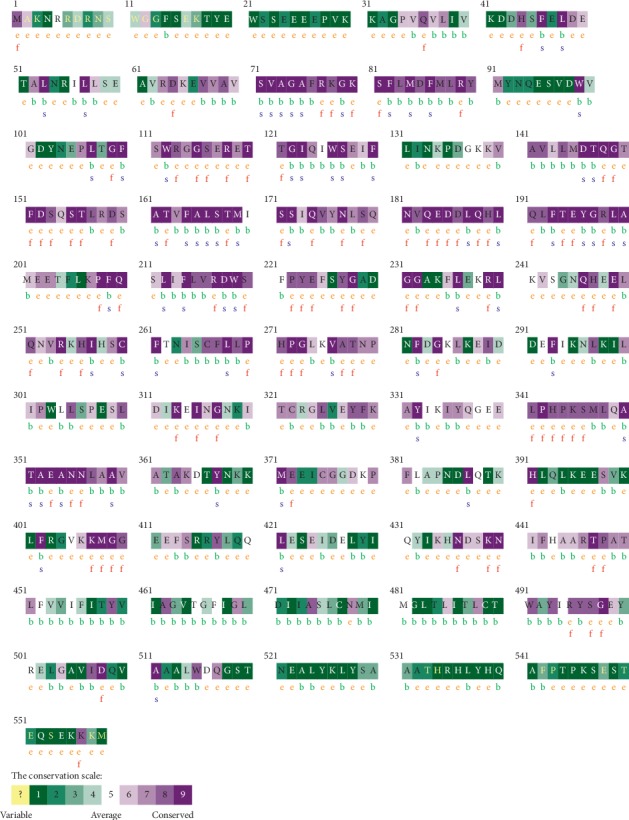
The conserved amino acids across species in ATL1 protein were determined using ConSurf. *e*: exposed residues according to the neural-network algorithm are indicated in orange letters. *b*: residues predicted to be buried are demonstrated via green letters. *f*: predicted functional residues (highly conserved and exposed) are indicated with red letters. *s*: predicted structural residues (highly conserved and buried) are demonstrated in blue letters. *I*: insufficient data (the calculation for this site performed in less than 10% of the sequences) is demonstrated in yellow letters.

We also used Consurf server to check the conservation region of ATL1 protein, and the result shows 4 SNPs (V67F, T120I, R217Q, and R495W) located in highly conserved regions, which can directly affect the protein function (Figure 10).

**Figure 11:**
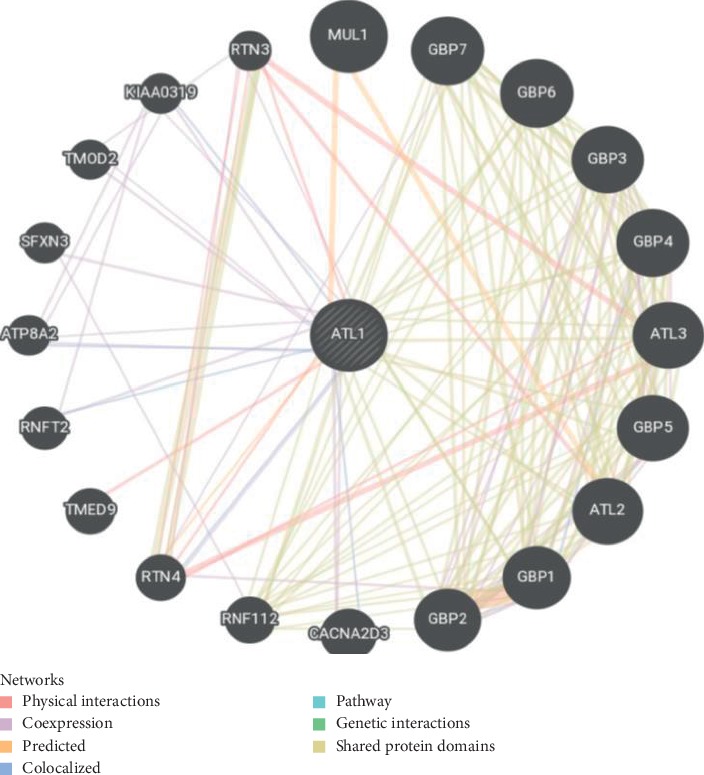
Biological network interaction between *ATL1* and its related genes.

We also used GeneMANIA, which showed that *ATL1* has many dynamic functions: endomembrane system organization, endoplasmic reticulum organization, and protein homooligomerization. The genes coexpressed with, sharing similar proteindomain, or contributed to achieve similar function as shown in (Table 5; Figure 11).

We also used ClinVar to compare our results that had been found by an in silico approach with the clinical one; in R217Q, SNP was found to be pathogenic, and our result does not match with this result [52]; however, some evidence-based studies match with our result [53, 52]. In the second SNP (R495W), the associated clinical studies in ClinVar show that our result matches with the reported record which is a pathogenic variant [54], while for the other SNPs (V67F, T120I, and G504E), we did not find any associated clinical studies.

**Figure 12:**
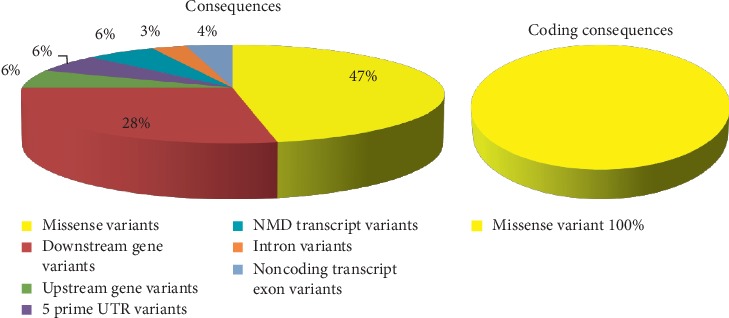
Summary pie charts and statistics.

The Variant Effect Predictor annotates mutations using an extensive array of reference data from previously detected mutations, evidence-based results, and estimation of biophysical consequences of mutations and that is what makes VEP an accurate web-based tool [45]. VEP described regulatory consequences for several mutations, including 15 mutations within a coding region, 15 mutations within a noncoding region, 2 mutations within upstream gene, 9 mutations within downstream gene, 1 mutation within noncoding transcript exon, and 2 mutations within 5 prime UTR variant; briefly, mutations within a coding region affect the protein function, while mutations within noncoding regions can significantly affect disease and could be contribute in the phenotypic feature and RNA-binding proteins (RBPs) [55, 56], while mutations in the upstream, downstream, 5′-, and 3′-UTRs might affect the transcription or translation process [57]. The consequences are shown in Table 6, while Figure 12 demonstrates the summary pie charts and statistics.

This study is the first bioinformatics analysis while all other studies were in vivo and in vitro analyses [17, 19]. To conclude, 5 disease-causing mutations were recognized as the most pathogenic SNPs in the coding region of *ATL1* gene that may cause SPG3A, and therefore, it may be used as genomic biomarkers for SPG3A. Lastly, Wet lab techniques are suggested to backing these outcomes.

## 5. Conclusion

In this study the impact of nsSNPs in the *ATL1 gene* was investigated by various bioinformatics tools that revealed the presence of five deleterious SNPs (V67F, T120I, R217Q, R495W, and G504E), which have a functional impact on ATL1 protein and, therefore, can be used as genomic biomarkers specifically before 4 years of age; also, it may play a key role in pharmacogenomics by evaluating drug response for this disabling disease.

## Figures and Tables

**Table 1:** Pathogenic nsSNPs predicted by different online tools.

dbSNP rs#	SUB	SIFT prediction	Score	Polyphen prediction	Score	PROVEAN prediction	Score	SNAP2 prediction	Score
rs778130435	F46S	Affect	0	Probably damaging	1	Deleterious	−7.014	Effect	48
rs746927118	V67F	Affect	0	Probably damaging	1	Deleterious	−4.221	Effect	36
rs979765709	T120I	Affect	0	Probably damaging	1	Deleterious	−5.216	Effect	83
rs149031604	T147I	Affect	0	Probably damaging	1	Deleterious	−5.544	Effect	79
rs119476049	R217Q	Affect	0	Probably damaging	1	Deleterious	−3.749	Effect	95
rs1212638776	A359T	Affect	0	Probably damaging	1	Deleterious	−3.722	Effect	20
rs864622269	R495W	Affect	0	Probably damaging	1	Deleterious	−7.265	Effect	73
rs1242753115	G504E	Affect	0	Probably damaging	1	Deleterious	−7.659	Effect	78

**Table 2:** The most damaging SNPs predicted by different online tools.

dbSNP rs#	SUB	SNPs&GO prediction	RI	Probability	PHD-SNP prediction	RI	Probability	P-mut prediction	Score
rs746927118	V67F	Disease	1	0.559	Disease	7	0.827	Disease	0.88 (92%)
rs979765709	T120I	Disease	2	0.622	Disease	5	0.765	Disease	0.92 (94%)
rs119476049	R217Q	Disease	3	0.626	Disease	6	0.81	Disease	0.84 (90%)
rs864622269	R495W	Disease	5	0.744	Disease	8	0.921	Disease	0.79 (89%)
rs1242753115	G504E	Disease	1	0.557	Disease	7	0.85	Disease	0.73 (87%)

**Table 3:** Protein structural stability calculated using I-Mutant 3.0 and MUPro.

dbSNP rs#	SUB	SVM2 prediction effect	RI	DDG value prediction	MUPro prediction	Score
rs746927118	V67F	Decrease	8	−1.39 kcal/mol	Decrease	−0.88922
rs979765709	T120I	Increase	2	−0.08 kcal/mol	Decrease	−0.39384
rs119476049	R217Q	Decrease	9	−1.09 kcal/mol	Decrease	−0.93524
rs864622269	R495W	Decrease	3	−0.24 kcal/mol	Decrease	−1.07508
rs1242753115	G504E	Decrease	4	0.59 kcal/mol	Decrease	−0.64728

**Table 4:** Shows evaluation of residues predicted by Ramachandran server.

Evaluation of residues
Residue [ 17:CYS] (−133.21, −153.02) in allowed region
Residue [ 25:GLN] (−162.85, 100.89) in allowed region
Residue [ 36:ARG] (−124.01, 73.57) in allowed region
Residue [ 58:ALA] (166.51, 154.01) in allowed region
Residue [ 79:SER] (73.74, 30.24) in allowed region
Residue [ 173:ALA] (−95.50, −67.11) in allowed region
Residue [ 176:GLY] (140.69, −38.32) in allowed region
Residue [ 186:GLY] (−78.37, −76.34) in allowed region
Residue [ 211:SER] (−160.66,−165.13) in allowed region
Residue [ 238:GLY] (88.32, −86.88) in allowed region
Residue [ 280:ASP] (52.68, −148.30) in allowed region
Residue [ 342:SER] (−130.57, 59.78) in allowed region
Residue [ 354:LEU] (64.56, −164.16) in allowed region
Residue [ 390:ASP] (−94.22, −161.52) in allowed region
Residue [ 410:ASP] (−110.37, −161.27) in allowed region
Residue [ 448:GLU] (−76.96, −29.27) in allowed region
Residue [ 451:PRO] (−58.25, 176.45) in allowed region
Residue [ 452:ARG] (−125.67, −177.93) in allowed region
Residue [ 496:PRO] (−37.48, 143.81) in allowed region
Residue [ 529:ASN] (−110.72, 55.22) in allowed region
Residue [ 535:LYS] (−165.28, 122.68) in allowed region
Residue [ 536:ASP] (57.02, −165.51) in allowed region
Residue [ 619:SER] (−110.89, −134.13) in allowed region
Residue [ 676:ASP] (54.03, −141.38) in allowed region
Residue [ 702:LEU] (75.96, −14.74) in allowed region
Residue [ 38:ASN] (−26.25, 134.98) in outlier region
Residue [ 64:SER] (173.42, 126.07) in outlier region
Residue [ 172:VAL] (−79.71, −93.88) in outlier region
Residue [ 239:ARG] (−35.14, 148.96) in outlier region
Residue [ 446:ARG] (75.93, 129.75) in outlier region
Residue [ 447:PRO] (−136.52, −173.35) in outlier region
Residue [ 455:THR] (165.16, 175.45) in outlier region
Residue [ 456:THR] (−98.45, 58.74) in outlier region
Number of residues in the favoured region (∼98.0% expected): 633 (95.0%)
Number of residues in the allowed region (∼2.0% expected): 25 (3.8%)
Number of residues in the outlier region: 8 (1.2%)

**Table 5:** The *ALT1* gene functions and its appearance in network and genome.

Function	FDR	Genes in network	Genes in genome
Endoplasmic reticulum organization	1.91E-07	5	19
Endomembrane system organization	0.0006584	6	210
Golgi organization	0.0956682	3	45
Cellular response to interferon-gamma	0.5758816	3	90
Response to interferon-gamma	0.7482479	3	106
Protein homo-oligomerization	0.8135839	3	116

FDR: false discovery rate; it is greater than or equal to the probability that this is a false positive.

**Table 6:** Shows variant consequences, transcripts, and regulatory features by VEP tool.

rs variations	Consequence	Gene SYMBOL	Feature	Protein position	Amino acids
rs746927118	Missense variant	*ATL1*	ENST00000358385.10	67	V/F
rs746927118	Missense variant	*ATL1*	ENST00000441560.6	67	V/F
rs746927118	Downstream gene variant	*ATL1*	ENST00000553509.1	—	—
rs746927118	Upstream gene variant	*ATL1*	ENST00000553746.1	—	—
rs746927118	Intron variant	*ATL1*	ENST00000554886.1	—	—
rs746927118	Missense variant	*ATL1*	ENST00000555960.5	67	V/F
rs746927118	Downstream gene variant	*ATL1*	ENST00000556478.2	—	—
rs746927118	5 prime UTR variant	*ATL1*	ENST00000557735.1	—	—
rs979765709	Missense variant	*ATL1*	ENST00000358385.10	120	T/I
rs979765709	Missense variant	*ATL1*	ENST00000441560.6	120	T/I
rs979765709	Downstream gene variant	*ATL1*	ENST00000553509.1	—	—
rs979765709	Noncoding transcript exon variant	*ATL1*	ENST00000553746.1	—	—
rs979765709	5 prime UTR variant	*ATL1*	ENST00000554886.1	—	—
rs979765709	Downstream gene variant	*ATL1*	ENST00000555960.5	—	—
rs979765709	Downstream gene variant	*ATL1*	ENST00000556478.2	—	—
rs979765709	Missense variant	*ATL1*	ENST00000557735.1	37	T/I
rs119476049	Missense variant	*ATL1*	ENST00000358385.10	217	R/Q
rs119476049	Missense variant	*ATL1*	ENST00000441560.6	217	R/Q
rs119476049	Missense variant	*ATL1*	ENST00000554886.1	73	R/Q
rs119476049	Upstream gene variant	*ATL1*	ENST00000555266.1	—	—
rs864622269	Missense variant	*ATL1*	ENST00000358385.10	495	R/W
rs864622269	Missense variant	*ATL1*	ENST00000441560.6	495	R/W
rs864622269	Downstream gene variant	*ATL1*	ENST00000555266.1	—	—
rs864622269	Downstream gene variant	*SAV1*	ENST00000555720.5	—	—
rs864622269	Missense variant, NMD transcript variant	*ATL1*	ENST00000556067.1	77	R/W
rs1242753115	Missense variant	*ATL1*	ENST00000358385.10	504	G/E
rs1242753115	Missense variant	*ATL1*	ENST00000441560.6	504	G/E
rs1242753115	Downstream gene variant	*ATL1*	ENST00000555266.1	—	—
rs1242753115	Downstream gene variant	*SAV1*	ENST00000555720.5	—	—
rs1242753115	Missense variant, NMD transcript variant	*ATL1*	ENST00000556067.1	86	G/E

## Data Availability

All data underlying the results are available as part of the article, and no additional source data were required.
